# The human malaria-*Aotus* monkey model: a historical perspective in antimalarial chemotherapy research at the Gorgas Memorial Laboratory-Panama

**DOI:** 10.1128/aac.00338-24

**Published:** 2024-06-05

**Authors:** Nicanor Obaldía

**Affiliations:** 1 Center for the Evaluation of Antimalarial Drugs and Vaccines, Instituto Conmemorativo Gorgas de Estudios de la Salud, Panama, Republic of Panama; 2 Department of Immunology and Infectious Diseases, Harvard University T.H. Chan School of Public Health, Boston, Massachusetts, USA; The Children's Hospital of Philadelphia, Philadelphia, Pennsylvania, USA

**Keywords:** malaria, antimalarials, chemotherapy, *Plasmodium falciparum*, *Plasmodium vivax*, *Aotus*, non-human primates, animal models, history of medicine, Panama

## Abstract

The human malaria-*Aotus* monkey model has served the malaria research community since its inception in 1966 at the Gorgas Memorial Laboratory (GML) in Panama. Spanning over five decades, this model has been instrumental in evaluating the *in vivo* efficacy and pharmacokinetics of a wide array of candidate antimalarial drugs, whether used singly or in combination. The animal model could be infected with drug-resistant and susceptible *Plasmodium falciparum* and *Plasmodium vivax* strains that follow a characteristic and reproducible course of infection, remarkably like human untreated and treated infections. Over the years, the model has enabled the evaluation of several synthetic and semisynthetic endoperoxides, for instance, artelinic acid, artesunate, artemether, arteether, and artemisone. These compounds have been evaluated alone and in combination with long-acting partner drugs, commonly referred to as artemisinin-based combination therapies, which are recommended as first-line treatment against uncomplicated malaria. Further, the model has also supported the evaluation of the primaquine analog tafenoquine against blood stages of *P. vivax*, contributing to its progression to clinical trials and eventual approval. Besides, the *P. falciparum*/*Aotus* model at GML has also played a pivotal role in exploring the biology, immunology, and pathogenesis of malaria and in the characterization of drug-resistant *P. falciparum* and *P. vivax* strains. This minireview offers a historical overview of the most significant contributions made by the Panamanian owl monkey (*Aotus lemurinus lemurinus*) to malaria chemotherapy research.

## INTRODUCTION

Malaria is ranked alongside tuberculosis and HIV/AIDS, collectively referred to as the “Big Three” (1), as one of the most important infectious diseases in the world. The disease transmitted to humans by the bites of infected female *Anopheles* mosquitoes is caused by five species of protozoan parasites of the genus *Plasmodium*, namely *Plasmodium falciparum*, *Plasmodium vivax*, *Plasmodium ovale*, *Plasmodium malariae*, and *Plasmodium knowlesi*.

Between 2000 and 2015, there was a notable decline of over 18% in malaria cases and 37% in the incidence of the disease worldwide (2). However, despite these advances, about 249 million new cases were reported in endemic countries in 2022, an increase of 5 million cases with an estimated 608,000 deaths compared to 2021 (3–6), the vast majority in children under 5 years old from sub-Saharan Africa (7). Compounding this problem, resistance to Qinghaosu (artemisinin) (QHS)—artemisinin-based combination therapies (ACTs)—is expanding globally (8), presenting a significant challenge to malaria elimination efforts.

The dramatic decrease in the morbidity and mortality observed in some countries in Africa and Asia during this period (9, 10) resulted in malaria no longer being the leading cause of death among children from sub-Saharan Africa (4), but recently, this decrease has stalled, hampering elimination efforts (3). This significant achievement was partly attributed to using fixed-dose ACTs as first-line treatment for uncomplicated *P. falciparum* malaria (11) and to the massive use of insecticide-treated bed nets (12).

However, QHS resistance, initially detected on the Thai-Cambodian border in the mid-2000s (13, 14), is now prevalent in Southeast Asia (15–19), South America, Papua New Guinea, and Eastern Africa (20), representing a serious threat to the global elimination effort (21, 22). Meanwhile, *Anopheles* mosquitoes continue to develop resistance to insecticides (23–25), and the reemergence of malaria in regions where it has been eliminated—attributable to complacency, inadequate commitment, insufficient funding, and global warming or climate change (26)—is a growing concern (27, 28).

Several ACTs are currently being used globally as the first-line treatment for uncomplicated malaria, and plans are in place to deploy triple combination therapies to tackle the rise of resistance to partner drugs and QHS in East Africa, which poses a significant threat to the gains at reducing the malaria burden in this region (20, 29–31).

The search for new, more potent antimalarials to combat multiple drug-resistant (MDR) strains requires improved pre-clinical models for their screening and evaluation.

This review summarizes the historical contribution of the Panamanian owl monkey (*Aotus l. lemurinus*), human *P. falciparum*, and *P. vivax* model to antimalarial drug chemotherapy research at the Gorgas Memorial Laboratory (GML) in Panama.

## THE SEARCH FOR A SUITABLE HUMAN MALARIA NHP MODEL

Before the advent of *P. falciparum in vitro* culture in 1976 by William Trager and James B. Jensen (32), human volunteers (33), malaria therapy hospitals that treated neurosyphilis patients (34–36), and non-human primates (NHP) were the only reliable sources of well-characterized *P. falciparum* and *P. vivax* parasites available for biological and antimalarial drug efficacy studies. Thus far, *P. vivax* cannot be cultured long-term *in vitro* because the parasite preferentially invades young reticulocytes (37).

Interest in malaria in NHP has captivated investigators at GML in Panama since its establishment in 1928 in honor of US Army General William Crawford Gorgas, a renowned physician for his work in combating yellow fever and malaria in Cuba and later in Panama during the construction of the Panama Canal (38). During the early days of the laboratory (1930–1931), Herbert C. Clark surveyed Panamanian monkeys in search of malaria parasites and found what he described as tertian malaria parasites resembling *P. vivax* in red spider monkeys (*Ateles geoffroyi*), and quartan malaria parasites resembling *P. malariae* in white face monkeys (*Cebus capucinus*) (39). Later, with Lawrence H. Dunn, he attempted to transfer monkey malaria parasites from spider monkeys (*Ateles* sp.) to humans with negative results (40). In 1934, William H. Taliaferro and Paul R. Cannon, working at GML, successfully transmitted *P. falciparum* to Panamanian howler monkeys (*Alouatta palliata)* (41–43).

In 1961, Martin D. Young and Donald V. Moore reported the first evidence of *P. falciparum* chloroquine (CQ) resistance from a man in Colombia (44). Thus far, no *P. falciparum* CQ resistance has been detected northwest of the Panama Canal (45–47). Later, resistance to amodiaquine (AQ) hydrochloride and hydroxychloroquine was reported from the same region (48). Meanwhile, almost simultaneously, resistance was spreading in Southeast Asia (49). These findings renewed the interest of scientists at GML in searching for a suitable NHP model that will allow them to study the biology of resistance and test the efficacy of new, more potent antimalarials to combat the spreading CQ resistance (50).

In 1963, the US Army Walter Reed Army Institute of Research (WRAIR) established an ambitious malaria research program to develop new drugs against infections with CQ-resistant (CQR) strains of *P. falciparum*. The program used avian, rodent, and simian malaria models to guide the synthesis of new agents and select compounds to test in human volunteers.

In 1966, in a landmark study, Martin D. Young, James A. Porter, and Carl Johnson, while working at GML in Panama, were able for the first time in medical research to transmit *P. vivax* from man to owl monkeys (*Aotus trivirgatus*) and back to man through the bites of infected mosquitoes. This groundbreaking experiment demonstrated that the *Aotus* monkey was a reliable malaria model (43, 51). Meanwhile, working at Stanford University, Geiman and Meagher developed the *P. falciparum Aotus* model (43, 52).

Hitherto, Leon Herbert Schmidt while working as founding director of the National Center for Primate Biology at the University of California at Davis since 1963 and later in 1969 at the Southern Research Institute in Birmingham, Alabama, until his retirement in 1976, when the project was transferred back to GML in Panama, characterized and validated the model using *Aotus* from northern Colombia (*Aotus lemurinus griseimembra)* (53–56). The studies aimed to leverage infections with human plasmodia in the search for new antimalarials effective against CQR strains of *P. falciparum*.

Initially, Schmidt investigated whether owl monkeys could be infected with *P. falciparum* and *P. vivax* strains that were being investigated in human volunteers and whether these infections were reproducible. Subsequently, he examined whether infections in the owl monkey responded to treatment with standard antimalarials in the same way as in humans. He also assessed whether pilot studies of new compounds could proceed with minimal use of experimental agents and animals (55).

The studies conducted by Schmidt characterized infections with *P. falciparum* and *P. vivax* and responses to standard antimalarials such as CQ, quinine (QN), and pyrimethamine (PYR) (55). These responses were highly comparable to those experienced by human volunteers or patients with malaria. Additionally, different blood schizontocidal responses were observed in infected *Aotus* monkeys when treated with experimental compounds of diverse chemical structures (56). This further illustrated and validated the value of the human *Plasmodium*-*Aotus* monkey model in the screening and selection of antimalarial drugs for clinical evaluation.

To investigate the similarity between the responses of infections in owl monkeys challenged with various test strains of *P. falciparum* and *P. vivax* and the responses in human volunteers or naturally infected patients to treatment, Schmidt (55) calculated the total course doses required to cure 50% and 90% of infections (CD90s and CD50s). He then compared these to the total milligram doses of each drug needed for uniformly curative results in an average 70-kg human subject infected with drug-susceptible strains, converting these into mg per kg doses. Subsequently, the mg per kg doses were converted to mg per square meter (m^2^) doses by multiplying the former by 10 for monkeys and 38 for humans.

As stated below by Schmidt in a review from 1969 (57) on the state of “chemotherapy of the drug-resistant malaria” that holds even today when referring to the discovery of the human malaria-*Aotus* model by Young, Porter, and Johnson in 1966 (51):

“This event has a number of extremely important implications for future developments in malaria therapy (apart from the remarkable opportunities that it affords for studies on the patho­physiology and immunology of infections with human plasmodia). In the first place, it makes possible for the first-time evaluation of the activities of potential new drugs against the same targets as are of concern to man. Secondly, it promotes systematic manipulations of pertinent drug-effect relationships against infections of diverse levels of development and severity that could never be approached in human volunteers, and only fortuitously and in an uncontrolled manner in the field. Lastly, it removes a heavy burden from human volunteer studies at a time when the number of subjects for any aspect of research medicine is limited, and the liabilities of exposing individuals to infections with drug-resistant malarias make for a precarious situation that is not always acceptable. The use of the owl monkey infected with the human malarias may well turn out to be a milestone in the search for new antimalarials” (57).

The human malaria-*Aotus* model, first reported at GML in Panama, made possible in the following decades, in addition to understanding the human malaria parasite biology, host–parasite–vector interactions (58, 59), immunology, and pathogenesis (60–65), allowed the development of *P. vivax ex vivo* cultures (37, 66), the adaptation, and cryo-preservation (67) of new drug-sensitive and MDR-resistant strains of *P. falciparum* (43, 68–71) and *P. vivax* (43, 72) for testing of new antimalarials and candidates vaccines (68, 73–83).

## THE HUMAN MALARIA-*AOTUS* MODEL AT GML IN PANAMA

The use of *Aotus l. lemurinus*, kariotypes VIII and IX (84–86), as a model to study the efficacy of antimalarial drugs has been ongoing without interruption at GML in Panama since 1976 under the aegis of WRAIR and other funding agencies and universities (87). This continuity is partly due to the availability of the model at GML, which still maintains a successful breeding program and self-sustainable *Aotus* colony (88–90), and the increasing drug resistance exhibited by highly pathogenic *P. falciparum* isolates from Asia (71), Africa, and Latin America and to the development of CQ resistance in *P. vivax* from the Melanesian and the Indonesian archipelagos (91), Vietnam (92), and South America (93).

Over the years, several strains of *P. falciparum* and *P. vivax* exhibiting diverse susceptibility and/or resistance to standard antimalarial agents (87) have been passaged or adapted to Panamanian *Aotus* monkeys. Many of these strains were initially adapted to *Aotus* and *Saimiri* monkeys by William E. Collins at the Centers for Disease Control and Prevention (CDC) in Atlanta, GA, USA, and further adapted to Panamanian *Aotus* or vice versa at GML in Panama by Richard N. Rossan (43). Among those adapted to Panamanian *Aotus* were the Vietnam Smith/RE, Vietnam Oak Knoll (FVO) (53), Uganda Palo Alto (52, 72), Indochina I (94), Malayan-Camp (77), Santa Lucia (68, 95), Nigeria (70), Honduras I (79, 82), Panama II (69, 96), and, more recently, the MDR Thai C2A clone (71). Additionally, several strains of *P. vivax* were adapted, including the Panamanian Achiote (72, 97), Santa Rosa strains (51), the New Guinea Chesson (CQ-sensitive), and AMRU-I (CQR) (91, 98), as well as CQ-sensitive Sal-I from El Salvador (62, 99). It is worth noting that the adaptation of human malaria field isolates to *Aotus* monkeys requires in excess of 20 serial passages, initially in splenectomized and further passages in spleen-intact animals (71, 94).

The course of untreated infections in Panamanian *Aotus* was characterized and compared with its close relative *Aotus l. griseimembra* from northern Colombia—considered by some as the most susceptible to malaria—by Rossan et al. in 1985 (100). Overall, in this study, they found that maximum parasitemias of the Vietnam Smith and Uganda Palo Alto strains were significantly higher during the first 15 days of patency in Panamanian than in Colombian owl monkeys, but during recrudescence, parasitemias were higher in Panamanian than in Colombian *Aotus*, though these strains appeared to be more pathogenic to Colombian *Aotus*. The differences observed were attributed to the geographic origins of monkeys and parasite strains (100).

Numerous candidate antimalarial drugs of diverse chemical classes have been evaluated against trophozoite-induced infections of one or more *P. falciparum* and *P. vivax* strains in this model since 1976 using the following protocol. The inoculation and follow-up with variations have been as follows: briefly, 1 mL of infected blood is drawn with 2.5% sodium citrate anticoagulant from the femoral vein of a donor monkey and diluted with incomplete RPMI tissue culture media or 0.85% saline solution to form a suspension of 5 × 10^6^ parasitized red blood cells/mL. One milliliter of the suspension is then injected into the saphenous vein of the experimental animals. Beginning with the first post-inoculation (PI) day, Giemsa-stained thick blood smears obtained from a prick made with a lancet in the marginal ear vein are prepared and examined daily, and parasitemia is determined using the Earle and Perez technique (101). By the fifth day, when parasitemia is about 5 × 10^3^ parasites/µL, the drug is administered orally or by parenteral routes. Doses are calculated on a mg base drug per kg basis. Response to treatment is then categorized as (i) none, when parasitemia in the treated subject is similar to the untreated control; (ii) suppressed, when parasitemia persists or is less than at the start of treatment or no greater than one-fiftieth the level of the control; (iii) cleared, when thick blood films are parasite-negative for at least 7 consecutive days before recrudescence; and (iv) cured, when thick blood films are negative during or immediately after treatment and remain negative during the follow-up period of 90 days (53, 71, 102).

A full discussion of each experimental antimalarial drug tested in *Aotus* monkeys at the GML throughout the years is beyond the scope of this minireview. Instead, the historical review will focus on selected compounds that have progressed to clinical trials or received approval.

## ANTIMALARIAL DRUG CLASSES

### Quinoline methanols

#### MQ (WR142490)

Discovered and developed commercially by the US Army Antimalarial Program and Hoffman La Roche during 1960–1970, mefloquine (MQ), a 4-quinolinemethanol analog of QN marketed as “Lariam,” to replace WR030090, an experimental antimalarial that demonstrated 88% cure rates and was better tolerated than QN in field trials, progressed into Phase II trials (103–105). MQ exhibits a long half-life and could be administered once a week for prophylaxis (106). Still, its neurologic side effects (psychiatric effects that could last years after use) limit its full potential (36). Nevertheless, today, it is being used alone or in combination with QHS derivatives as an effective ACT, except for areas of Southeast Asia, where MQ resistance is prevalent (107).

In 2006, Dow et al. (103) reported the antimalarial activity of less neurotoxic and more potent antimalarials of a number of 2-substituted alkylaminoquinoline methanols (AAQMs) related to WR030090 in Panamanian *Aotus* monkeys. In a preliminary experiment to benchmark the new antimalarials, MQ was administered to *P. falciparum* FVO-infected *Aotus* at single oral doses of 3.1, 6.25, 12.5, and 25 mg/kg on Day 4 PI, to determine the lowest curative dose that resulted in 6.25 mg/kg (94). In a subsequent experiment, the least neurotoxic AAQMs compounds were tested against *P. falciparum* FVO infections in *Aotus*. This time, WR069878 and WR035058 cured infections in *Aotus*, while WR074086 and WR176399 only cleared but recrudesced. Clinical failures were associated with a high *in vitro* IC90 (≥20 ng/mL) against *P. falciparum* TM91C235 and/or relatively low plasma concentrations of these compounds (103). Further experiments found WR069878 effective at clearing or curing CQR *P. vivax* AMRU-I and *P. falciparum* infections in *Aotus* at 10 mg/kg × 3 and 7 days, respectively. The compound was also more potent than MQ against MQ-resistant strains of *P. falciparum in vitro*.

In 2009, Obaldia et al. (71) adapted through serial passage to *Aotus* the Thai TM90C2A (C2A) multidrug-resistant *P. falciparum* clone originally isolated from a patient in Thailand in 1992, before the observation of altered susceptibility to QHS in Southeast Asia. This strain has demonstrated resistance to CQ, sulfadoxine–PYR (SP), QN, MQ, and the artesunate–MQ combination. During adaptation to *Aotus*, the parasite showed resistance to 20- or 40-mg/kg oral dose of MQ, and the infections were only cured when MQ was administered orally at 40 mg/kg in combination with intravenous (i.v.) artesunate (AS) at 20 mg/kg for 3 days. Similarly, the clone was only suppressed by artelinic acid (AL) at 8 and 16 mg/kg for 3 days, while QN at 20 mg/kg for 5 days had no effect or only suppressed in combination with the experimental dihydrofolate reductase (DHFR) inhibitor (WR297608) at 10, 20, or 40 mg/kg for 3 days, with atovaquone/proguanil (Malarone) at 25 mg/kg for 3 days clearing but recrudesced.

### Endoperoxides

#### Artemisinin (QHS)

In 2015, the Nobel Prize for Medicine or Physiology was shared by the Chinese scientist Tu Youyou for her contribution to the discovery of Qinghaosu (artemisinin) in 1972, working under project 523 formed in 1967 by the directive of the Chinese government (108, 109). This extremely active antimalarial produces the most rapid clearance of any antimalarial known (108). The medicinal herb Qinghao (*Artemisia annua* or sweet wormwood) has been used in China to treat intermittent fever for over 2,000 years (109). Eventually, oral ACTs entered into clinical trials and were adopted as the first-line treatment for uncomplicated malaria by WHO in 2006 (108, 110), while parenteral i.v. AS was indicated for severe, complicated malaria (107). By 2009, decreased susceptibility to AS was reported in western Cambodia, and since then, it has spread to the rest of Southeast Asia (107).

#### Tricyclic 1,2,4-trioxanes

Pre-clinical studies of these synthetic analogs of QHS in mice and NHP carried out by Posner et al. in 1994 demonstrated that tricyclic 1,2,4-trioxanes numbers 8 and 9 when administered intramuscularly (i.m.) at 12 mg/kg cleared parasitemia in each of two monkeys but recrudesced in one. Retreatment at 48 mg/kg and initial treatment at this dose was as effective as arteether (AE) at curing infections in *Aotus* monkeys of the MDR *P. falciparum* Vietnam/Smith strain. These synthetic trioxanes were relatively inexpensive and easily prepared and offered high hopes of preparing compounds with good oral bioavailability (111).

#### AM (WR254986AB) and AE (WR255131AE)

Derivatives of dihydroartemisinin (DHA) [artemether (AM) and AE] are effective against CQ-sensitive and CQR *P. falciparum* strains. In the early 1980s, the WHO selected AE over AM, arguing that the former, more lipophilic, would accumulate in brain tissues in patients with cerebral malaria and for its easy separation during synthesis (102).

In the early 1990s, Shmuklarsky et al. (102) carried out a series of experiments in three pre-clinical test systems, including *in vitro*, the *Plasmodium berghei* mouse test system, and the *P. falciparum*/*Aotus* monkey model (Table 1).

**TABLE 1:** *In vitro* concentration and treatment doses in mice and *Aotus* monkeys of selected artemisinin derivatives alone or in combination against infections with *P. berghei* and MDR *P. falciparum^a^*

	*In vitro*	Mice			*Aotus*					
	*P. falciparum*	*P. berghei*			*P. falciparum*	Rx				
Compound	IC50	CD50	mg/kg/day/once	Outcome	Strain	mg/kg	days	Route	Outcome	Reference
Artemisinin (QHS)	4.11*									102
Artemether (AM)	1.74*	55		C	Vietnam Smith/RE	7.1	1	i.m.	C, R	102
Arteether (AE)	1.61*	55		C	Vietnam Smith/RE	11.8	1	i.m.	C, R	102
Artesunate (AS)					FVO	8	3	i.v.	C, R	114
					C2A	8, 20	3	i.v.	S or CL, R	68
Artelinic acid (AL)					C2A	8, 16	3	po	S	68
Artemisone (ASO)					FVO	10	3	po	CL, R	120
AS + mefloquine (MQ)					C2A	8, 40	3, 1	i.v., po	C	68
ASO + MQ					FVO	10 + 5–12.5	1	po	C	120
MQ					FVO	6.25	1	po	C	91
					C2A	20, 40	1	po	S, CL, R	68

^*a*^ *, H3-hypoxanthine assay; C, cured; CD50, 50% curative dose; CL, cleared; IC50, 50% inhibitory concentration; i.m., intramuscular; i.v., intravenous; MDR, multiple drug resistant; po, oral; R, recrudesce; Rx, treatment; S, suppressed.

Approximately 60 monkeys were used in these experiments. The animals were treated i.m. in three equal doses at 12-hour intervals starting on the fifth day PI with both drugs dissolved in sesame oil (USP). Typically, treated monkeys showed a rapid decrease in parasitemia of the *P. falciparum* Vietnam Smith/RE strain after 1–2 days of treatment. By 1 week, the parasites were by or under the detection limit of fewer than 10 parasites per µL. The clearance time was about 7–8 days for both compounds. At 48- to 192-mg/kg dosages, all infections were cured, while at lower doses, all recrudesced. The 50% effective curative dose was approximately 7 mg/kg for AM and 12 mg/kg for AE, both being about 2.5-fold more potent than QHS *in vitro*. The authors concluded that both compounds were equipotent (102). It is interesting to note that the ED50 of AM of 7. 1 mg/kg in the *Aotus* monkey was comparable to the clinically effective curative dose of AM in humans of 8.6–12 mg/kg (in a 50-kg human receiving a dose of 480–600 mg i.m. in divided doses for several days). Later, both drugs were approved for use in humans (AM with lumefantrine, marketed as Coartem) and artheeter injection to treat uncomplicated *P. falciparum* malaria (112).

#### Artesunate (AS) (WR256283)

Intravenous AS is the preferred treatment for severe malaria. In a series of experiments to test the efficacy of i.v. AS alone at 8 mg/kg per 3 days in spleen-intact Panamanian *Aotus* monkeys inoculated with the CQR *P. falciparum* FVO strain, all monkeys treated cleared their infection but recrudesced (113). The monkeys were retreated with the same dose regimens between Days 10 and 15 post-treatment (PT) but needed rescue treatment with MQ.

Intrigued by the decreased susceptibility to AS observed during the adaptation of the MDR Thai *P. falciparum* C2A strain when administered orally to *Aotus* at 33 mg/kg per 3 days, Obaldia et al. (107) investigated the efficacy of AS alone or in combination with MQ in six splenectomized *Aotus* monkeys. This study aimed to rule out the inoculum size effect (high vs low) reported by others during QHS susceptibility testing in laboratory strains *in vitro* (114). In these experiments, the animals were infected with a high *Aotus* passage (passage X) of the MDR Thai *P. falciparum* C2A clone, using a standard inoculum of 5 × 10^6^ parasites. This clone was isolated from a patient in a Thailand hospital in 1992, before the advent of QHS resistance in Southeast Asia (107). The MDR Thai *Aotus*-adapted *P. falciparum* C2A clone demonstrated resistance to orally administered MQ at doses as high as 40 mg/kg once and AS at 33 mg/kg per 3 days (fourfold higher than that needed to clear *P. falciparum* FVO infections in *Aotus*) alone or in combination and comparable to *in vitro* data obtained from parasites during adaptation (Table 1) (71, 107).

Furthermore, in this experiment, the combination of AS at 33 mg/kg per 3 days and MQ at 40 mg/kg orally failed to clear but cured when AS was administered i.v. at 20–33 mg/kg during re-treatment of the low parasitemia-low passage (III–IV) parasites (personal communication) (71). Further analysis, with *ex vivo* drug assays, demonstrated decreased susceptibility of the C2A clone to MQ, CQ, QHS, DHA, and AS in the high-passage (X), compared to low-passage (III) parasites. Even though neither mutations in the kelch K13 propeller domain associated with QHS resistance (115) nor changes in *Pfmdr1* copy number associated with MQ resistance were detected (116). These findings suggested that the mechanism of resistance observed was due to increased parasite fitness to the NHP host during adaptation (107) or to an alternative drug resistance mechanism, as others have suggested (117).

#### ASO (BAY 44-9585)

Artemisone (ASO), a highly active 10-alkylaminorate second-generation semi-synthetic QHS derivative developed by Bayer, Germany, for antimalarial therapy devoid of neurotoxicity (118), was tested by Obaldia et al. in 2009 against experimental infections with *P. falciparum* FVO in *Aotus* monkeys (119). During these experiments, when given once at 10 mg/kg of body weight, ASO cured *Aotus* monkeys in combination with MQ at 5 and 10 mg/kg, but not at 2.5-mg/kg single oral dose. When the compound was given orally at 10 mg/kg/day for 3 days with AQ at 20 mg/kg/day, ASO cured all infected monkeys. Similarly, ASO at 30 mg/kg/day plus clindamycin at 100 mg/kg/day was also curative (119). This compound went to human clinical trials in consortium with the Hong Kong University of Science and Technology, Kowloon, Hong Kong, and Medicines for Malaria Venture (MMV) (29).

### 4-aminoquinolines

#### CQ (WR1544BM)

While working at Bayer in Germany, Johann “Hans” Andersag, in 1934, modified quinacrine (marketed as mecaprine or quinacrine) by replacing its acridine ring with a quinoline ring and discovered a compound with high antimalarial activity (109, 120). The resulting compound named resochin and its derivative sontonchin, later to be rediscovered in 1945 by EK Marshal as CQ, unlike methylene blue (MB) or quinacrine, did not stain the skin and eyes (109, 120). CQ, an extraordinary antimalarial compound, was the first-line antimalarial treatment until about 20 years later when antimalarial drug resistance developed around the world (109, 120).

As mentioned above, by 1975, Rossan et al. (72), working at GML, had cured trophozoite and sporozoite-induced infections of the *P. vivax* Achiote strain in 12 *Aotus* monkeys with CQ at 25-mg base/kg body weight over a 3-day course alone in the trophozoite-induced infection and in combination with primaquine (PQ) at 1 mg base/kg during 14 days in sporozoite-induced infections, achieving radical cures, with parasite clearance occurring in both cases within 2–6 days after treatment. During these experiments, vivax relapses did occur after CQ was administered alone to *Aotus* and Saimiri monkeys in sporozoite-induced infections, indicating for the first time evidence for the persistence of exoerythrocytic stages of *P. vivax* in a New World NHP.

#### CQ resistance reversers

In 1987, reversal of CQ resistance in *P. falciparum*, was achieved *in vitro* by the co-administration of verapamil (a calcium channel blocker used in the treatment and prophylaxis of angina and therapy of supraventricular tachyarrhythmias) plus CQ (121). Other *in vitro* studies have shown that CQR *P. falciparum* parasitized erythrocytes accumulated significantly less CQ than CQ-sensitive falciparum parasites (122); this phenomenon was described as strikingly similar to that observed in the inhibition exerted by verapamil on the release of anticancer agents by multidrug-resistant mammalian cancer phenotypes. Raising hopes that the clinical response to CQ could be restored (123).

With this information on hand, in 1988, Bitonti et al.*,* using the tricyclic antidepressants desipramine and norpramin, demonstrated for the first time the feasibility of reversing CQ resistance *in vivo* using *Aotus* monkeys infected with the CQR *P. falciparum* Smith/RE strain. However, in each case, parasitemia recrudesced (124). In this study, desipramine was found to be the most effective compound to reverse CQ resistance *in vitro* and *in vivo* (124).

Later, in 1993, in another series of experiments, Kyle et al. (123, 125), using combinations of CQ plus antihistaminic drugs such as chlorpromazine, prochlorperazine, and promethazine, confirmed *in vivo* reversal of CQ resistance in *Aotus* monkeys infected with the CQR *P. falciparum* Vietnam Smith/RE strain. Describing an *in vivo* efficacy order for reversing CQ resistance in Panamanian *Aotus*, with chlorpromazine being more potent than prochlorperazine >>desipramine >> Ro 11–2933 (tiapamil analog) > ketotifen, with cyproheptadine and verapamil having no effect or displaying toxicity in combination with CQ (123).

In another study in 2018, Obaldia et al. (126) reversed CQ resistance in *Aotus* infected with the CQR *P. vivax* AMRU-I strain from Papua New Guinea, treated orally with CQ at 10 mg/kg and prochlorperazine—a drug used to treat nausea, migraines, schizophrenia, psychoses, and anxiety—at 20 mg/kg alone or in combination, for 5 consecutive days, whereas neither drug alone produced cures. This same drug combination reverses CQ resistance in *P. falciparum*, and the authors concluded that the combination could be an alternative for treatment in humans with CQR *P. vivax* infections.

### 8-aminoquinolines

#### PQ (WR2975AW)

In 1925, a team of chemists and biologists working at Bayer in Germany developed plasmoquine, the first 8-aminoquinoline synthesize based on the structure of QN with activity capable of preventing *P. vivax* relapses—now known to be caused by hypnozoites or dormant liver stages—and with remarkable potency against avian malaria (109, 127). Soon after World War II, the American effort trying to improve plasmoquine introduced PQ after comprehensive studies in the 1950s by Schmidt using the rhesus monkey animal model infected with *Plasmodium cynomolgi*, avoiding the continuation of clinical trials in human volunteers infected with falciparum or vivax malaria (127). Fifty years later, another PQ derivative, tafenoquine (TQ), a novel 8-aminoquinoline that emerged from the US Army Antimalarial Program, established at the WRAIR in 1963, in partnership with Glaxo-Smith-Kline (GSK) and MMV, was approved for human use by the Food and Drug Administration in 2018 (29, 91, 109, 128). Until then, PQ, which has serious side effects, including hemolysis in glucose-6-phosphate dehydrogenase-deficient individuals, was the only drug approved for the radical cure of *P. vivax* infections (127, 129).

As noted earlier, in 1975, Rossan et al. (72) showed the first chemotherapeutic evidence of the persistence of exoerythrocytic stages of *P. vivax* in New World monkeys. During these experiments, using sporozoite-induced infections of the *P. vivax* Panamanian *Achiote* strain in *Aotus* and Saimiri monkeys, they were able to detect relapses ranging from 38 to 111 days after treatment with CQ at 25-mg base/kg body weight over 3 days or a single dose of 10 mg base/kg in three of 11 Saimiri and three of five *Aotus* and radically cure it with CQ at 25 mg base/kg plus PQ at 1 mg base/kg for 14 days (72).

#### TQ (WR238605)

This novel 8-aminoquinoline PQ analog developed at WRAIR (127), first in humans in 1998 (130), promised a radical cure of *P. vivax* infections (131) without most of the side effects of PQ and has since proved to be an effective prophylactic antimalarial as well (132–134). In the late 1990s, TQ was evaluated in *Aotus* monkeys against the *P. vivax* AMRU-I CQR strain from Papua New Guinea (135). In 1997, Obaldia et al. (136), using TQ, CQ, and their combinations against trophozoite-induced infections of the AMRU-I strain in *Aotus* monkeys, demonstrated that TQ alone at a total dose of 9 mg/kg over a 3-day course cured all three monkeys of their infections. In contrast, a total dose of 30–60 mg/kg of CQ did not clear patent parasitemia or clear but recrudesced. Total doses of 30 mg/kg of CQ or 3 mg/kg of TQ alone failed to cure, yet both drugs given in combination at these dosages cured two of three infections. These results indicated that TQ, currently marketed as “Krintafel” by GSK combined with CQ, is an alternative treatment for CQR vivax malaria.

### Phenothiazines

#### Methylene blue

In the late 1800s, Paul Ehrlich, a highly renowned German Scientist-Physician using MB to stain malaria parasites (120), noticed that the parasites took the stain avidly, postulating that if used *in vivo*, it might poison and kill the parasite. To test his hypothesis, in 1891, he used it to cure two patients of malaria, the first time a synthetic drug—magic bullet—was used in humans. Until then, the only available treatment for malaria was QN extracted from the bark of the South American Cinchona tree (109).

Antimalarial trials with MB continue at Bayer in Germany, which found MB to be less effective than QN. Nevertheless, Röhl, a disciple of Paul Ehrlich who used birds to test antimalarials, designed a new compound from MB by replacing a methyl group with an aminoalkyl group. This led scientists at Bayer to eventually synthesize “quinacrine” in 1931, the precursor of CQ (120).

More than 100 years later, looking for alternatives to treating severe malaria by the parental route, Ohrt et al. (113), in a series of experiments, tested MB orally or i.v. in *Aotus* monkeys infected with the *P. falciparum* FVO CQR strain. During pilot experiments, MB (USP) administered orally to *Aotus* monkeys did not cure infections, presumably to low bioavailability. However, when MB was administered i.v., all doses studied (8, 16, and 24 mg/kg) cleared infections by the fifth day after the beginning of therapy but recrudesced.

No clear dose-response was detected in these experiments, presumably because all doses were at the top of the dose-response curve. Compared with historical AS controls, MB cleared parasitemia somewhat slower on the first day of treatment but was similar on Days 2 and 3. The clearance day mean value was approximately equal to both MB and AS, though MB was more effective in delaying recrudescence than AS. In this study, one animal in the AS 8 mg/kg group presented severe anemia on day 50 PT that appeared not to result from a sub-patent infection (113). Delayed anemias have also been reported in children in the second and third week after i.v. AS treatment and adults with complicated malaria (137, 138).

### DHFR inhibitors

PS-15, a new antifolate belonging to the oxyguanils class of drugs, metabolized *in vivo* to WR99210, an extremely active triazine inhibitor of DHFR abandoned by the Army Antimalarial Drug Program because of gastrointestinal intolerance and poor bioavailability during clinical development. Based on an analogy to the metabolism of the biguanide proguanil to the triazine cycloguanil, the biguanide precursor for the triazine WR99210 was designed and synthesized. In 1993, Canfield et al. (139) administered proguanil, cycloguanil, and PS-l5 orally to *Aotus* monkeys infected with the Vietnam Smith/RE or Monterrey strain of *P. falciparum* (both strains are resistant to CQ, QN, and PYR). Neither proguanil nor cycloguanil was curative at the doses tested in these experiments. However, both PS-15 and WR99210 were extremely active, with PS-15 being more active than WR99210.

### Transition state analog inhibitors of PNP

#### DADMe-immucillin-G (BCX4945)

Purine salvage in *P. falciparum* relies on hypoxanthine salvage and can be disrupted with transition-state analog inhibitors effective against both human and *Plasmodium* purine nucleoside phosphorylases (PNPs). Blocking PNP kills cultured malaria parasites by purine starvation. DADMe-Immucillin-G (BCX4945) is a transition state analog of human and *Plasmodium* PNPs. In 2011, Cassera et al. (140) found that when the PNPs BCX4945 was administered orally at 50 mg/kg twice a day for 7 days to *P. falciparum* FVO-infected *Aotus* monkeys, parasitemia was cleared between Days 4 and 7 of treatment, remaining negative for up to 9 days PT when recrudescence occurred. During the 30-day trial, no signs of toxicity were observed in the monkeys. Blood samples from an infected untreated monkey showed increased PNP activity as the parasitemia increased, while treated animals showed approximately 98% PNP inhibition during the treatment period. These studies indicated that oral administration of BCX4945 to *Aotus* for 7 days resulted in parasite clearance and recrudescence in an otherwise *P. falciparum* lethal infection in *Aotus* monkeys. Like QHS, the clearance and recrudescence were likely to the short half-life in plasma exhibited by this drug and to the high levels of hypoxanthine found in the plasma and blood cells of *Aotus* (plasma: mean = 40 mM; blood cells: mean = 64 mM), when compared to plasma hypoxanthine in humans reported to be 2.7 mM. The authors concluded that further studies with this compound were warranted using a partner drug.

### Antibiotics

#### Azithromycin

Experiments carried out by Andersen et al. in 1995 (141) in Panamanian *Aotus* demonstrated that azithromycin, a semi-synthetic acid-stable macrolide antibiotic analog of erythromycin, discovered in Croatia in 1980 by the pharmaceutical company Pliva (142), when administered orally to monkeys infected with the CQR *P. falciparum* Vietnam Smith/RE strain at a dose of 100 mg/kg for 7 days, cleared and cured parasitemias on Days 10–14 PT (143), while azithromycin or doxycycline at 30 mg/kg for 7 days only suppressed or cleared but recrudesced. Comparatively, MQ typically clears and cures parasitemia of the CQ *P. falciparum* FVO strain by the fourth day after therapy at 20 mg/kg orally once in *Aotus* monkeys (94). Consequently, researchers have considered azithromycin alone or in combination to prevent or treat uncomplicated falciparum malaria in humans (142).

## CONCLUSIONS

Due to their physiological and immunological similarities with humans, NHPs remain a valuable animal model for research. Initially chosen for ethical reasons and the lack of *in vitro* culture, their relevance is also underscored by their approximation to human drug bioavailability, distribution, and metabolism. The information obtained from this animal model has undoubtedly played an essential role in protecting human subjects before advancing compounds to clinical trials.

In the past 60 years, regulations such as the Animal Welfare Act of 1966 (144), the first federal law in the USA to regulate the treatment of animals in research, and the European Directive 2010/63/EU on the protection of animals for scientific purposes that emphasizes the principles of the 3Rs—Replacement, Reduction, and Refinement, including housing and care as well as a psychological well-being plan for primates—had tightened restrictions on the use of NHP. The directives also encouraged the development of alternative research methods that do not involve animals, such as computer modeling, tissue engineering, and cell culture techniques (145).

Despite the emergence of the controlled human malaria infection challenge models (33, 146), based upon *in vitro* and *in vivo* mouse model efficacy and pharmacokinetics data in rats (146), and the development of the humanized mouse models for studying malaria blood (147–150) and hepatic stages (151), these innovative approaches require further evaluation to ascertain its efficacy fully. The human *Plasmodium*/*Aotus* model remains, to this date, an invaluable laboratory animal that still plays an essential role in the search for new antimalarial drugs and vaccine candidates to help eliminate this scourge of humanity.
